# Editorial: Neurostereology

**DOI:** 10.3389/fnana.2019.00042

**Published:** 2019-04-16

**Authors:** Bente Pakkenberg, Mikkel Vestergaard Olesen, Sanne Simone Kaalund, Karl-Anton Dorph-Petersen

**Affiliations:** ^1^Research Laboratory for Stereology and Neuroscience, Bispebjerg Hospital, Copenhagen, Denmark; ^2^Department of Clinical Medicine, Faculty of Health Sciences, University of Copenhagen, Copenhagen, Denmark; ^3^Translational Neuropsychiatry Unit, Department of Clinical Medicine, Aarhus University, Aarhus, Denmark; ^4^Centre for Stochastic Geometry and Advanced Bioimaging, Aarhus University, Aarhus, Denmark; ^5^Translational Neuroscience Program, Department of Psychiatry, University of Pittsburgh, Pittsburgh, PA, United States

**Keywords:** brain, stereological methods, disector, fractionator, cavalieri volume

Quantification of cells in a three-dimensional (3-D) structure requires robust estimates based on unbiased principles. Studies using two-dimensional methods (2-D) to report data of 3-D structures are still frequently used. However, these methods do not consider the irregular nature of biological structures in terms of shape and distribution of the target features. Consequently, they make assumptions about the 3-D structure of interest and the results do not refer to the complete structure. These limitations reduce the sensitivity and accuracy of the methods as well as increase the risk of errors. This can be avoided by the application of design-unbiased stereology. Stereology is based on a set of statistical and mathematical principles and provides efficient tools for estimation of volume, surface area, length, and number of objects in 3-D structures by sampling in 2-D sections. Because stereology relies on statistical sampling principles and stochastic geometric theory, it is guaranteed that no methodological biases are introduced to the analysis. Thus, in principle, it allows one to obtain accurate and precise quantitative data of structural changes in biological tissue.

In this special volume, the papers will focus on the application of different stereological methods and their practical aspects. The papers are introduced and explained by prominent neurostereologists and reported in the order by which *the method* was first introduced.

The first paper (Basler et al.) deals with the precision of the Cavalieri estimator of volume. If correctly applied, the resulting sampling-generated variability should not be able to mask significant group differences. To provide tentative answers to the question if sampling has been “good enough,” the authors discuss the influence of sampling frequency, smoothness factor and section orientation on the Gundersen-Jensen coefficient of error (CE). Using the layers of the mouse hippocampal dentate gyrus as an example they found that the CE provided reasonable estimates of the precision obtained using different sample sizes. The data are presented, allowing the reader to approximate sampling intervals in frontal, horizontal, or sagittal sections that provide CE's of specified sizes.

The second paper by Fichtl et al. uses intact, macro- and microscopically well-preserved postnatal human cerebellar hemispheres allowing for high-precision morphologic investigations. The study identifies anatomically distinct cerebellar fissures and delineate functionally relevant regions. It also describes how to estimate the volume of regions of interest and quotes the literature for proven sampling schemes. The paper is richly illustrated.

The physical disector method and its use in the industry is described in the paper by Fabricius et al. The study describes how automated alignment of microscopic images allows for efficient stereological analysis of specific dopaminergic neurons in the substantia nigra of hemiparkinsonian rats. The authors conclude that the automated physical disector provides a useful and efficient tool for unbiased estimation of selected cell types in regions of interest in the rat brain.

Napper describes the use of the optical disector and its application to the rodent brain using immunohistochemistry. It emphasizes that estimates of numerical density can result in misleading data, most often in an unknown direction. The author shows how new developments in electron microscopy enable the application of design-based stereology, particularly the disector method, to this type of sections. In the study serial block-face scanning electron microscopy is used to efficiently obtain total number data at an ultrastructural level.

The study by Larsen describes the use of the optical fractionator applied to the human fetal brain. Simple estimates of cell volumes and densities may be unreliable due to unpredictable shrinking artifacts, and the fragility of e.g., the fetal brain requires particular care in histological handling and processing. Aiming at just total numbers, the optical fractionator design is especially useful and offers direct, robust, and reliable estimates.

Kreutz and Barger gives an example of the optical fractionator using immunofluorescence techniques. Improvements in immunohistochemistry and fluorescence imaging technologies have facilitated easy application of immunofluorescence protocols, allowing for visualization of multiple target proteins in one tissue sample. Combining immunofluorescence labeling with stereological data collection can thus provide a powerful tool to maximize explanatory power and efficiency, while minimizing tissue use. The paper provides a protocol for reliably integrating the optical fractionator technique and multiple immunofluorescence techniques.

Parker and Sweet provide a review of dendritic spine density, number of cells, and application of the nucleator to provide pyramidal cell somal volume in auditory cortex. They identify and describe potential neural substrates for auditory impairment and gray matter loss in the auditory cortex in schizophrenia. The review highlights how stereology has been crucial for obtaining proper data collection, reporting and, ultimately, interpretation of the complex relationship between the target estimates.

West describes the practical application of the space ball probe and reviews its use in a number of studies focusing on axon, dendrite, and capillary length in the nervous system. The review provides a discussion of the salient features of the methodology of length, the validity of the method and details potential difficulties in its application to histological tissue.

Finally, Boyce and Gundersen complete this special volume on neurostereology by describing the application of the automatic proportionator for estimation of a sparse cell populations. The proportionator, an estimator based on non-uniform sampling theory, marries automated image analysis with stereological principles. It provides a highly efficient and precise method to address the challenge of quantitating e.g., sparse cell populations in the central and peripheral nervous system in situations where traditional stereological methods based upon systematic, uniformly random sampling are impractical. The power of the proportionator as a stereological tool is illustrated.

## Author Contributions

BP, MVO, SSK, and K-AD-P collaborated together in initiating this Topic, and in writing the editorial.

### Conflict of Interest Statement

The authors declare that the research was conducted in the absence of any commercial or financial relationships that could be construed as a potential conflict of interest.

